# Drug delivery nanocarriers and recent advances ventured to improve therapeutic efficacy against osteosarcoma: an overview

**DOI:** 10.1186/s43046-021-00059-3

**Published:** 2021-02-08

**Authors:** Sujit Arun Desai, Arehalli Manjappa, Preeti Khulbe

**Affiliations:** 1 School of Pharmacy, Suresh Gyan Vihar University, Mahal Rd, Mahal, Jagatpura, Jaipur, Rajasthan 302017 India; 2 Annasaheb Dange College of D Pharmacy, Ashta, Tal: Walwa, Dist., Sangli, Maharashtra 416301 India; 3 Tatyasaheb Kore College of Pharmacy, Warananagar, Tal: Panhala, Dist., Kolhapur, Maharashtra 416113 India

**Keywords:** Osteosarcoma, Nanocarriers, Stimuli-responsive nanocarriers, Active targeting, Gene therapy, T cell therapy

## Abstract

**Background:**

Osteosarcoma (OS) is one of the key cancers affecting the bone tissues, primarily occurred in children and adolescence. Recently, chemotherapy followed by surgery and then post-operative adjuvant chemotherapy is widely used for the treatment of OS. However, the lack of selectivity and sensitivity to tumor cells, the development of multi-drug resistance (MDR), and dangerous side effects have restricted the use of chemotherapeutics.

**Main body:**

There is an unmet need for novel drug delivery strategies for effective treatment and management of OS. Advances in nanotechnology have led to momentous progress in the design of tumor-targeted drug delivery nanocarriers (NCs) as well as functionalized smart NCs to achieve targeting and to treat OS effectively. The present review summarizes the drug delivery challenges in OS, and how organic nanoparticulate approaches are useful in overcoming barriers will be explained. The present review describes the various organic nanoparticulate approaches such as conventional nanocarriers, stimuli-responsive NCs, and ligand-based active targeting strategies tested against OS. The drug conjugates prepared with copolymer and ligand having bone affinity, and advanced promising approaches such as gene therapy, gene-directed enzyme prodrug therapy, and T cell therapy tested against OS along with their reported limitations are also briefed in this review.

**Conclusion:**

The nanoparticulate drugs, drug conjugates, and advanced therapies such as gene therapy, and T cell therapy have promising and potential application in the effective treatment of OS. However, many of the above approaches are still at the preclinical stage, and there is a long transitional period before their clinical application.

## Background

Of the many bone cancers, osteosarcoma (OS) is the most general prime malignant bone tumor accounting for 60% [1]. Both children and adults between 10 and 20 years of age are affected by OS. OS is a complex unbalanced karyotype tumor having some chromosomal aberrations. Although a variety of genetic factors has been correlated with OS, the specific cause of the OS is not known. Pain is one of the frequent symptoms of OS.

Recently, chemotherapy followed by surgery and then post-operative adjuvant chemotherapy is the widely used conventional strategies for OS treatment. However, the clinical applications of most of the chemotherapeutics have been limited due to lack of selectivity and sensitivity to tumor cells, toxicity towards normal cells, multi-drug resistance (MDR), poor pharmacokinetic performance and, etc. [2, 3]. Furthermore, lower blood flow to the bone also acts as a barrier (blood-bone marrow barrier) in the delivery of anti-tumor therapeutics to the bone [4]. Therefore, there is an unmet need to develop novel and multi-functional strategies for the effective treatment of OS.

The administration of two or more anticancer drugs in combination is found to be more effective than a single therapeutic due to their multiple pathway action. Besides, these combination approaches are able to reduce the MDR; however, the selective targeting of drugs to the bone is also essential to treat OS effectively. Different nanotechnology-based drug delivery systems including nanoparticles, micelles, liposomes, dendrimers, nanogels, etc. have been developed to tackle the limitations of conventional chemotherapy [5]. These nanocarriers (NCs) can improve drug solubility, selectivity to tumor cells, permeability by enhanced permeability and retention (EPR) effect and lengthen circulation time. Moreover, these nanosystems can release the drug in response to specific stimuli such as pH, temperature, magnetism, and ultrasound [6, 7]. Among a variety of nanoparticles (NPs), biogenic calcium carbonate is found to be promising because of its better biocompatibility, slow biodegradability, pH-sensitivity, and osteoconductivity [8, 9].

Both passive and active targeting strategies can be effective to target the therapeutic at the bone. The specific targeting of carrier-drug conjugates to various tumors can be achieved by conjugation of specific carriers or ligands with drugs. The various targeting ligands such as Bisphosphonates (BP), N-(2-hydroxypropyl)-methacrylamide (HPMA), and tetracycline (TC) are found to be potential in the bone targeting and treating metastatic cancers due to their high affinity towards hydroxyapatite (HA) [10–12]. Nowadays, gene therapy is a promising option to improve the performance of existed therapy. Gene therapy is effective to treat complex diseases like OS that are related to genetic defects. Genetically modified T cell therapy is another advanced approach used to treat the OS.

In this current review, we discuss the case studies of organic nanoparticles tested against OS including modified nanoparticle approaches developed to further improve the efficacy against OS. In addition, the drug conjugates prepared by chemically conjugating drugs with copolymers and/or ligands which having a high affinity towards bone were explained with available case studies. Besides, advanced promising approaches such as gene therapy, gene-directed enzyme prodrug therapy, and T cell therapy tested against OS are also briefed.

## Main text

### Drug delivery challenges in OS and other cancer types

The drug delivery to OS is very challenging because of the presence of various barriers such as unrevealed etiology, huge histological diversification, big genomic instability, deficiency of definite biomarkers, abundant local aggressiveness, fast blood clearance, and a quick metastasizing potential [13]. The other general barriers associated with solid tumors include tumor microenvironment, tumors associated with vasculature, and stroma cells.

The tumor microenvironment plays a crucial role in the drug delivery to tumors including solid tumors. Heterogeneity of the tumor microenvironment is one of the significant barriers limiting drug accumulation by inducing drug resistance. The distinctive properties of solid tumors compared to normal tissues include abnormal vascular network (non-functional vessels and shunting of blood) [14], collective solid strain because of the quick tumor growth, and raised interstitial fluid pressure (IFP) owing to augmented permeability of the tumor-associated vasculature and the lack of functional lymphatic drainage. The increased IFP is one of the key barriers that cause blood flow stasis and reversed blood flow, which hamper drug uptake by tumor regions. Besides, another main barrier is the overproduction of proteins from the extracellular matrix leads to solid stress and compressed tumor vessels [15]. These barriers in combination with the perfusion defects cause hypoxia and acidosis, with necrotic and non-perfused tumor regions making cancer cells more resistant towards chemotherapeutics.

In the case of tumors-associated vasculature, the endothelial cell lining of blood vessels is surrounded by pericytes and a basal membrane. The endothelial cell lining acts as an important barrier between the underlying tissue and the blood that affects chemotherapeutic delivery [16, 17]. Moreover, in the tumor cells, uncontrolled neovascularization will take place resulting in a lack of hierarchical branching organization [13]. The vessels are unevenly dilated causing disordered tumor blood flow and can even alter the directions. Besides, tumor vessels do not mature completely because of rapid growth and pre-dominance of vascular endothelial growth factors (VEGF). The gaps (leaky vasculature) can be observed between vascular lining because of the lack of tight arrangement between the pericytes and endothelial cells [13, 18]. This leaky vasculature with an ineffective lymphatic system causes increased IFP thereby reducing drug uptake [19].

Tumor stromal cells also act as one of the significant barriers in drug delivery to solid tumors. These stromal cells are involved in tumor progression, promote tumor vascular bed, and can protect the tumor from attacks by the immune system. In addition to all above, the lower blood flow to the bone (blood-bone marrow barrier) also poses a challenge for efficient delivery of therapeutics to the bone [4].

### Approaches used to improve therapeutic performance in OS

The three main approaches, according to the American Cancer Society, applicable for OS treatment are surgery, chemotherapy, and radiotherapy. Initially, surgical resection was the widely used treatment strategy for OS however greater than 80% of patients afterward developed a recurrent disease that typically presented as pulmonary metastases [20]. Therefore, systemic chemotherapy after surgery was found to be of vital importance in OS [21]. Various anticancer drugs like doxorubicin (DOX), cisplatin, and ifosfamide were tried for treating OS however none have shown effective and satisfactory treatment because of drug resistance and adverse effects of a high dose of anticancer therapeutics [22–24]. Therefore, a drug combination approach is implemented to defeat the above problems. The combination of edelfosine with DOX in OS has been reported to exhibit a synergistic therapeutic effect. Also, edelfosine is able to reduce the dose of DOX administration. However, it is observed that the combined drug administration in the free form can suffer to achieve uniform concentrations at the site of action as a result of their independent kinetic and dynamic profiles to achieve a synergistic effect. Therefore, there is a need to develop novel strategies to impart uniform kinetic and dynamic characteristics to all drugs used in combination therapy which in turn further improves the therapeutic performance of the combination therapy in OS [25].

### Nanoparticulate drug delivery

Nanomedicines are found to be one of the promising approaches for the effective treatment of cancers. The nanocarriers can improve the biopharmaceutical properties of drugs, targeting efficiency, pharmacokinetic and dynamic performance, in vivo stability, control release, and diminish the side effects [26]. Both small drug molecules and macromolecules can be effectively targeted to the bone using a nanoparticle approach. A variety of nanocarriers such as liposomes, polymeric NPs, gold NPs, quantum dots, injectable hydrogels, nanogels, metallic nanoparticles (NPs), solid lipid NPs, dendrimers, or albumin NPs, micelles, and micelleplexes have been developed for treating osteosarcoma effectively [26–31].

**Fig. 1:**
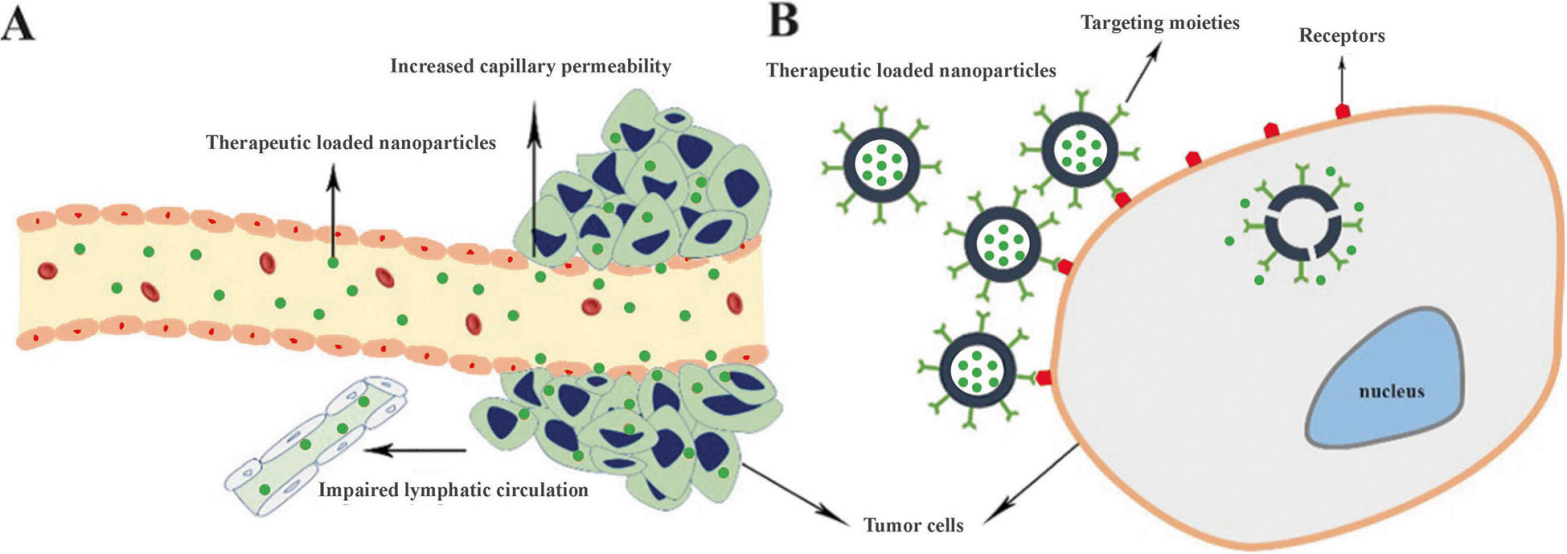
Targeting mechanisms of nanocarriers against cancer. **a** Passive targeting where NCs accumulated in tumors as a result of leaky blood vessels and impaired lymphatic drainage in tumor tissues (EPR effect). **b** Active targeting wherein NCs surface decorated with ligands interact with cancer cells and internalized via receptor-mediated endocytosis mechanism [32]

These nanocarriers are mainly taken up by macrophages, mononuclear phagocytes reticulo-endothelial system (RES), and inflammatory tissues. These NCs do not undergo extravasation into healthy tissues as a result of tight endothelial junction of capillary blood vessels. However, in many solid tumors, these NCs undergo efficient extravasation and retain in the tumor interstitium as a result of leaky vasculature and poor lymphatic drainage (the EPR effect) (Fig. 1a). The bone marrow contains extremely fenestrated capillaries with pore sizes up to 170 nm in diameter [32]. To accumulate therapeutics at tumor sites, NCs should be long-circulating, and it can be achieved by modifying NCs surface with hydrophilic polymers such as polyethylene glycol (PEGylation). Moreover, NCs of 50–100 nm in size can enter the parenchymal hepatic cells whereas the NCs with size < 50 nm can enter the spleen and bone by penetrating through the endothelial cells of the liver or through the lymph. Thus, the reduction of NCs size is having great importance in avoiding NCs intake by the liver and increasing their distribution in the bone.

The diverse NPs fabricated against OS are listed in Table 1, and same are briefed here. Suliman and coworkers fabricated catechins modified selenium (Se) doped HA NPs (C-Se-HA NPs) with the intention of reducing the toxicity of Se to normal and stem cells. C-Se-HA NPs showed significant antitumor activity against human OS MNNG/HOS cells through the generation of reactive oxygen species [33]. To reduce MDR, Yagmur and co-workers have developed liposomes co-loaded with hydrophilic gemcitabine (GCB) and lipophilic clofazimine (CFM). They reported remarkable cytotoxicity with the developed liposomes as a result of the synergistic effect of GCB and CFM [34]. Similarly, to improving DOX efficiency against OS by reducing the P-glycoprotein (P-gp) mediated efflux of DOX and its cardiotoxicity, the HA-conjugated DOX liposomes (HCDL) containing H_2_S releasing fraction were developed by Elena and group. The presence of H_2_S releasing fraction caused a substantially increased effect against tumors overexpressed with P-gp, and also resulted in decreased cardiotoxicity [35]. In another study, Yanhai and the group have developed the curcumin (CUR)-loaded alendronate-hyaluronic acid octadecanoic acid (amphiphilic material) micelles (CAHM). They revealed improved solubility, bioavailability, and targeting of CUR towards OS as a result of increased affinity between the HA and CD44 receptors [36].

**Table 1:** Colloidal NPs tested for the treatment of osteosarcoma

Type of nanocarrier	Drug	Performance	Reference
HA NPs	Catechins	In vitro: significantly improved cellular uptake and antitumor activity against human osteosarcoma MNNG/HOS cells.	[33]
Liposomes	GCB and CFM	In vitro: significant (*p* < 0.05) cytotoxicity against Saos-2 cells than plain combination of GCB and CFM, and alone.	[34]
Liposomes	DOX	In vitro: increased cytotoxicity against U-2OS and U-2OS/DX580 than plain DOX and marketed DOX Caelyx. In vivo: significantly (*p* < 0.05) enhanced tumor growth inhibition in female Balb/C than plain DOX and marketed DOX Caelyx.	[35]
Micelles	CUR	In vitro: improved cytotoxicity against MG-63 cells when compared to free CUR.	[36]
PDPN	SHK	In vitro: increased cytotoxicity against 143B cells than plain SHK. In vivo: significant (*p* < 0.001) reduction in tumor volume by and pulmonary metastasis in BALB/c nude mice than plain SHK.	[37]
Polymeric NPs	CUR	In vitro: remarkable cytotoxicity than plain CUR against 143B cells.	[38]
Nanoliposomes	DOX	In vitro: improved cytotoxicity against Saos-2 cells than non-targeted liposomes and plain DOX.	[39]
Chitosan conjugated PLGA NPs	DTX and ALD	In vitro: sustained release of DTX and ALD, improved cellular uptake, and cytotoxicity against MG-63 cells.	[40]
Dendrimers	pTRAIL	In vitro: improved transfection efficacy on osteosarcoma MG-63 cells than commercial transfection reagents.	[41]

Suoyuan and the group have developed a peptide-decorated disulfide-crosslinked polypeptide nanogel (PDPN) with the objective of improving the intracellular delivery and reducing the systemic toxicity of medicinal herb extract shikonin (SHK) in sarcoma-targeting. The SHK delivered via PDPN has shown minimum systemic toxicity than plain SHK [37]. To tackle issues such as poor solubility, stability, and cellular uptake associated with CUR, Guanyi and coworkers have fabricated the CUR-loaded polymeric NPs. The nanoparticulate CUR exhibited improved solubility, stability, and cellular uptake in OS treatment. Moreover, the nanoparticulate CUR has exhibited remarkable in vitro cytotoxicity when compared to plain CUR [38]. The targeted nanoliposomes containing DOX payload and surface decorated with peptide have shown substantially higher toxicity against Saos-2 OS cells when compared to normal bone cells. Further, these targeted nanoliposomes have shown 1.5-fold and 1.91-fold higher cytotoxicity than non-targeted liposomes and plain DOX, respectively [39].

To improve the therapeutic efficacy against OS, Docetaxel (DTX) and Alendronate (ALD) were incorporated into chitosan conjugated PLGA NPs. These NPs, prepared by nanoprecipitation technique, are found to within the tumor targeting range (~ 200 nm) and displayed effective positive charge (20 mV) capable to increase cellular uptake efficiency [40]. Yu and groups have designed the triazine-modified dendrimers for efficient tumor necrosis factor (TNF)-related apoptosis-inducing ligand (TRAIL) gene therapy in OS. The triazine-modification caused improved therapeutic efficacy of TRAIL gene therapy against OS [41].

### Stimuli-responsive nanocarriers

The stimuli-responsive nanocarriers are useful to avoid premature drug release. The two strategies used for designing stimuli-responsive drug delivery systems include endogenous and exogenous stimuli. Endogenous stimuli are also known as biological or internal stimuli where specific internal factors present in the tumor microenvironment or inside cancer cells including enzymes, low pH, ATP, glutathione level, redox-potential, and hypoxia, etc., could be precise triggers for controlled drug release, endosome/lysosome escape, prodrug activation, tumor-specific imaging and therapy [42]. For designing this type of delivery system, specific materials should be selected to achieve respond to definite endogenous stimuli that cause structure disruption of nanocarriers, which results in the abrupt release of the enclosed drug. The exogenous (external) stimuli such as thermal, magnetic field, electronic field, ultrasound, and light can cause structural disruption of the nanocarriers that result in drug release at targeted tissue [43, 44]. The chief benefits of these stimuli are location and intensity of stimuli can be controlled, it is possible to add or remove external stimuli as per requirement, the multi-function performance in cancer theranostics can be obtained by employing multiple external stimuli, and stimuli for hours to days can be given. However, for particular metastatic lesions where the location is not certain, external stimuli would be impractical. Recently, researchers are focusing on the combination of stimuli-responsive system to achieve effective targeting in a variety of cancers [43, 45, 46].

The diverse stimuli-responsive nanocarriers reported against OS are listed in Table 2, and same are described here. Ting and group have fabricated hyaluronidase-responsive multi-layer liposome (HRML) co-loaded with cisplatin and Nrf2 siRNA (siNrf2) for targeting osteosarcoma. The HRML showed significant inhibition of tumor growth in the xenograft osteosarcoma mice with the least systemic side effects [52].

**Table 2:** The reported stimuli-responsive nanocarriers for the treatment of OS

Type of nanocarrier	Drug	Type of stimuli	Performance	Reference
NPs	DOX and PTX	reduction/pH	In vitro: significantly higher cytotoxicity against K7 cells than plain DOX NPs and PTX NPs, and free DOX and PTX.	[47]
Hydrogel	MTX and ALD	Thermosensitive	In vitro: sustained release of both MTX and ALD. In vivo: significant tumor inhibition activity in mice.	[48]
CeO2NPs	DOX	pH sensitive	In vitro: exhibited pH dependent sustained and controlled release of DOX.	[49]
Liposomes	DOX	Reduction and (GSH) sensitive	In vitro: remarkable cytotoxicity to MG63 OS cells than LO2 liver cells.	[50]
Hydrogel	PLK1-shRNA and DOX	Thermosensitive	In vitro: remarkable cytotoxicity than single drug-loaded hydrogels.	[51]

Significantly higher glucose consumption and lactic acid accumulation are observed in the tumor due to extensive metabolism. Thus, in the tumor micro-environment, the acidic endolysosomes (pH 5.0–6.0) and the reductive cytosol (containing 2–10 mM GSH) are found to be ideal for the development of stimuli-responsive (redox and pH) nanomedicines due to their major differences from the extracellular environment. Yongshuang et al. have developed dual (reduction and pH)-responsive NPs co-loaded with doxorubicin (DOX) and paclitaxel (PTX) (DOX-PTX NPs) for OS treatment. In this study, PEGylated poly (α-lipoic acid) copolymer (mPEG-PαLA) was utilized an amphiphilic and dual responsive material. The developed NPs have shown increased cytotoxicity and are attributed to the synergistic effect of DOX and PTX [47]. The thermosensitive hydrogel is a special type of injectable biomaterial which is in the solution state at low temperature that causes improved drug loading. When the mixed solutions are injected into the tumor site, get quickly converted into the gel state due to stimulation of body temperature that results in slow release of drug from the hydrogel. Hongli and group have developed new injectable methotrexate (MTX) and alendronate (ALD) co-loaded thermo-sensitive hydrogel for local delivery against OS. The observed synergistic anticancer effect with MTX and ALD present in thermo-sensitive hydrogel [48]. The microparticle delivery system loaded with cerium dioxide (CeO2) nanoparticles (< 25 nm) and the anticancer drug DOX was fabricated by Christos and co-workers. The developed microparticles are pH sensitive and are made of calcium carbonate and collagen type I. The microparticle system has displayed pH-dependent sustained and controlled release of DOX. Besides, microparticles showed increased chemotherapeutic action against the osteosarcoma cell line SAOS-2, and with reduced toxicity against the heart myoblastic cell line H9C2. At pH 6.0, the synergic effect of the pro-oxidant CeO2 nanoparticles and of the encapsulated doxorubicin leads to almost 100% of cell death [49].

Xuelei and coworkers developed the surface of the cationic and glutathione-responsive liposome functionalized with estrogen (ES) for targeted delivery of DOX in osteosarcoma. The naturally biocompatible chotooligosaccharides (COS, MW2-5 KDa) were covalently attached to the liposomal surface through a disulfate bond (-SS-) to confer reduction-responsive COS detachment, whereas estrogen was grafted via polyethylene glycol (PEG 2 K) chain to achieve estrogen receptor-targeting. The liposomes were stable in physiological conditions but rapidly released the DOX in response to tumoral intracellular glutathione (20 mM). Overall, the developed system showed promising results against estrogen receptor expressing OS [50].

The biodegradable, thermosensitive, and injectable PLGA-PEG-PLGA hydrogels were developed by Ma and group for localized co-delivery of PLK1shRNA and DOX against OS. The strategy of localized, sustained co-delivery of PLK1shRNA and DOX by using the biodegradable, injectable hydrogel in vivo led to almost complete suppression of tumor growth up to 16 days, significantly enhanced PLK1 silencing, higher apoptosis of tumor masses, as well as increased cell cycle regulation [51].

### Targeting strategies developed against OS

Depending upon the target, the strategies are mainly classified into organ-targeted, cell-targeted, and molecular-targeted therapy. In organ-targeted therapy, the drug is delivered to a specific organ where it accumulated in high concentration. Of the different bone compositions, hydroxyapatite (HA) is an important one. Most of the calcium available in the human body is in the form of HA. HA is a highly biocompatible material commonly used in bone repair. Currently, it is observed that HA nanoparticles (HANPs) are effective in the suppression of the growth of different cancer cells [53]. Victoria et al. have developed HANPs loaded with bisphosphonate medronate (bone-targeting moiety) and bromodomain inhibitor drug JQ1 (a chemotherapeutic agent). The in vitro activity of these NPs was evaluated in 2D and 3D K7M2 OS in vitro models. The 2D culture assays of medronate and JQ1-loaded HANPs showed inhibition of OS cell migration from the tumor spheroids [54].

In cell-targeted therapy, the substances like nucleic acid (NA) or proteins are delivered into specific cells. Nowadays, aptamer-based (single-stranded DNA and RNA oligonucleotides or polypeptide fragments) tumor-targeted therapy is found to be a promising approach [55]. The aptamers act directly on extracellular targets; hence, they are usually used in combination with anti-cancer drugs to target tumor cell surfaces. The important characteristic of the aptamer is its ability to accurate reorganization of tumor cells. Therefore, in the future, it is expected that aptamers recognizing and binding osteosarcoma cells could be identified and used for the diagnosis and treatment of osteosarcoma. Wang and co-workers have designed a novel aptamer using the cell-based systematic evolution of ligands exponential enrichment (cell-SELEX) technique that particularly recognizes the human osteosarcoma cell line (U-2 OS) [56].

In molecular-targeted therapy, the cell fusion and phagocytosis processes are utilized to deliver specific molecules such as proteins, peptides, or gene products. The protein molecules or gene segments related to particular cancers are the main targets of molecular therapy. The therapeutics can bind selectively to the target sites that cause tumor cell death. The angiogenesis and pulmonary metastasis are observed to be chief targets for osteosarcoma [57]. Dubois et al. have used anti-angiogenesis therapy (sunitinib) for advanced osteosarcoma treatment [58].

### Active targeting of therapeutics and their nanoparticulate forms

**Fig. 2:**
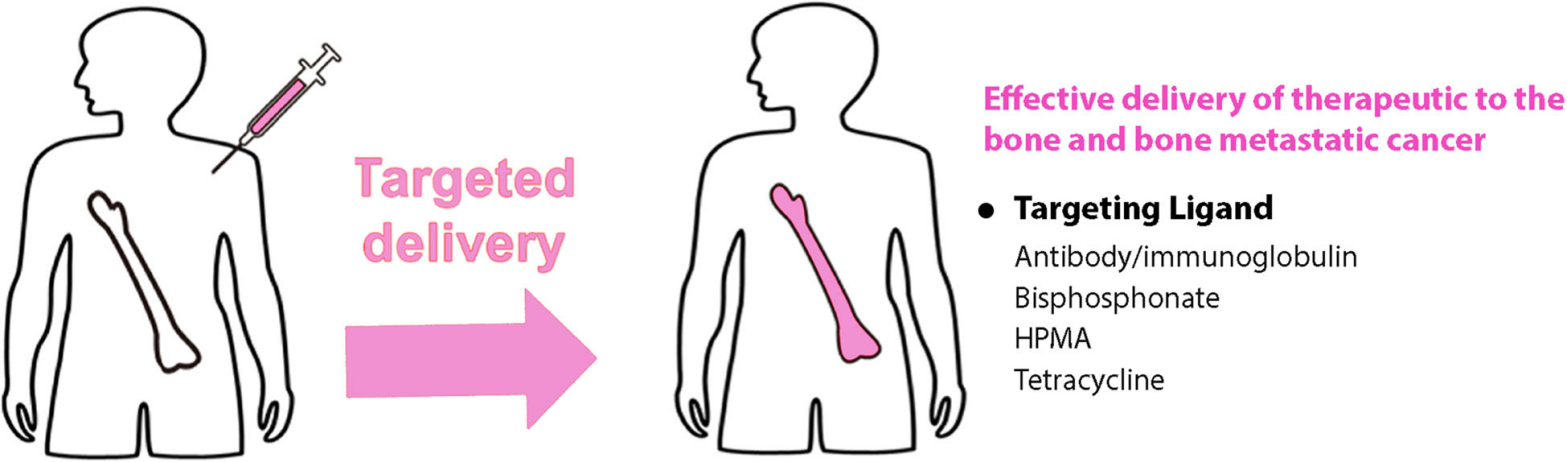
Targeted delivery of drug and nanoparticles to OS by conjugating ligands and polymers (such as antibodies, BP, HPMA, and tetracycline) having high affinity towards bone

Active targeting can be achieved by using targeting moieties such as specific ligands attached to drug molecules. Moreover, the surface of NPs can be decorated to achieve the active targeting of payloads (Fig. 1b). The molecules that bind specifically to the HA are used as therapeutics carrier (ligands) in bone targeting. The substances like Bisphosphonates (BP), diphosphonic acid, tetracyclines, propylene acid, heterocyclic small molecule, monoclonal antibodies, and oligopeptides have a high affinity for HA and hence can be used as carriers (ligands) in bone targeting of drugs or nanoparticulate-drugs [59–62] (Fig. 2).

The diverse actively targeted nanocarriers developed against OS are presented in Table 3, and the same are summarized here. Gui et al. developed all-trans retinoic acid (ATRA) NPs conjugated with CD133 aptamer (Apt-ATRA NPs) for targeting OS. Apt-ATRA NPs exhibited notable anti-tumor efficacy than plain ATRA and ATRA NPs without aptamer [63]. In another study, Chi and co-workers developed DOX-loaded redox-sensitive and HA functionalized (DOX-RHA) liposomes with improved anti-tumor efficacy against OS by targeting CD44 receptors. The liposomes displayed long circulation half-life and GSH-triggered cytoplasmic release of DOX [64]. Fang and co-workers designed Arg-Gly-Asp (RGD)-terminated poly(ethylene glycol)-block-poly (trimethylene carbonate)-targeted polymeric micelles (PMs) loaded with DOX (RGD-DOX PMs) to achieve better anti-cancer efficacy of DOX against OS. The micelles showed better cellular uptake due to the presence of RGD [65]. The LC09 (OS cell-specific aptamer)-functionalized PEG-PEI-Cholesterol (PPC) lipopolymer encapsulating CRISPR/Cas9 plasmids encoding VEGFA gRNA and Cas9 (LC09-PPC-CRISPR/Cas9) NPs have been developed for OS targeting. The LC09-PPC-CRISPR/Cas9 NPs showed higher in vitro cellular uptake than PPC-CRISPR/Cas9. Besides, NPs displayed increased accumulation of LC09-PPC-CRISPR/Cas9 in vivo in OS tissues and metastatic OS tissues in the lung of the mice compared to PPC-CRISPR/Cas9 [66]. DOX-conjugated BP NPs exhibited 40% more inhibition of tumor growth in a mouse bearing Saos-2 human osteosarcoma xenograft when compared to free DOX. The NPs showed improved circulation half-life because of the presence of polyethylene glycol (PEG) [67]. Ni and groups prepared salinomycin-loaded PEGylated poly(lactic-co-glycolic acid) NPs (SAL-NP) conjugated with CD133 aptamers (Apt-SAL-NP) using the solvent evaporation technique. These NPs showed a potent anti-tumor effect against OS by targeting CD133^+^ which is known as the best cancer stem cells (CSCs) marker of OS [68]. Morton et al. studied that tissue-targeted layer by layer (LBL) NPs loaded with DOX and coated with alendronate is observed to bind and internalize rapidly in human osteosarcoma 143B cells. Besides, liposomes showed significantly improved, prolonged tumor accumulation and efficacy of DOX in nude mice bearing 143B xenografts [69].

**Table 3:** Active targeted NPs reported for osteosarcoma treatment

Type of nanocarrier	Targeting moiety	Drug	Performance	Reference
Lipid-polymer NPs	CD133 apt	ATRA	In vitro: significant (*p* < 0.01) cytotoxicity towards Saos-2 CD133+ cells than apt-ATRA NPs and free ATRA. In vivo: substantially reduced tumor volume in BALB/c nude mice bearing osteosarcoma xenograft.	[63]
Liposomes	HA	DOX	In vitro: significantly (*p* < 0.01) higher cytotoxicity towards MG63 cells than NRS and NHA liposomes. In vivo: efficient tumor suppression in MG63 xenograft mouse model than NRS and NHA liposomes.	[64]
PMs	RGD	DOX	In vitro: remarkable cytotoxicity against MG-63 and MNNG/HOS OS cells than non-targeted DOX PMs.	[65]
LC09-PPC-CRISPR/Cas9 NPs	LC09 aptamers	CRISPR/Cas9 plasmids encoding VEGFAgRNA and Cas9	In vitro: enhanced cellular uptake than PPC-CRISPR/Cas9. In vivo: improved accumulation of LC09-PPC-CRISPR/Cas9 NPs in OS tissues and metastatic OS tissues in lung of the mice.	[66]
BP nanoparticles	BP	DOX	In vivo: enhanced anti-tumor efficacy in mouse bearing Saos-2 human OS xenograft than free DOX.	[67]
Polymeric NPs	CD133 apt	SAL	In vitro: increased cytotoxicity to Saos-2 CD133^+^ and U-2 OSCD133^+^ cells than SAL-NP and free SAL.	[68]
LbL liposomes	Alendronate	DOX	In vivo: improved anti-tumor efficacy in nude mice bearing 143B xenografts.	[69]

### Challenges for nanoparticulate drug delivery in OS and other cancer types

The scalable, controlled and reproducible manufacturing of nanoparticles under good manufacturing practice (GMP) conditions presents unique challenges [70]. The changes in the raw materials and small modifications in the manufacturing process can result in significant changes in the physicochemical properties (size, shape, composition, crystallinity, drug loading and release, and surface functionality and chemistry, etc.) of nanoparticles. These physicochemical alterations eventually influence the biological outcomes of the nanomedicines. In addition, finding the suitable methods to describe the physicochemical or biological properties of nanoparticles is challenging from a technical as well as a regulatory standpoint.

Despite the incredible advancement in NCs for OS treatment, still there are certain challenges that need to defeat. NCs that target tumors passively via the EPR effect are associated with certain drawbacks including inefficient drug diffusion into tumor cells, the random nature of targeting, and the lack of EPR effect in some tumors. The applications of active targeting by certain ligands have been limited because of various issues. For instance, in the case of active targeting by BP, they target the bone better than OS. Moreover, the prolonged residence of BP in the bone tissue may inhibit osteoclasts and bone homeostasis [71].

Nanoparticles, when compared with the same material at the larger scales, display significantly high surface area to volume ratio. Therefore, nanoparticles show altered interactions with cells and biomolecules, biodistribution profile, and safety profile. The long-term effect of nanoparticles on human health is not fully understood; as a result, the intense regulatory processes of clinical trials are set forth by the regulatory agencies. Besides, the nanoparticles require rigorous approval process for human use, and the clinical trials require huge amount of money. Taking all these issues into account, the extensive research is needed to identify the possible toxicities of nanomedicines before their clinical use.

Although many nanomedicine are available in the market, the manufacturing, characterization at biophysical levels, and clinical application of nanoparticulate drugs (complex therapeutics) has hampered due to unavailability of specific regulatory guidelines. Therefore, the regulatory organizations must develop complete regulations required for manufacturing and complete list of tests that cover the physicochemical and biological (efficacy, biodistribution and toxicity) characterization of nanoparticles.

### Drug conjugates

#### Immunoconjugates

**Fig. 3:**
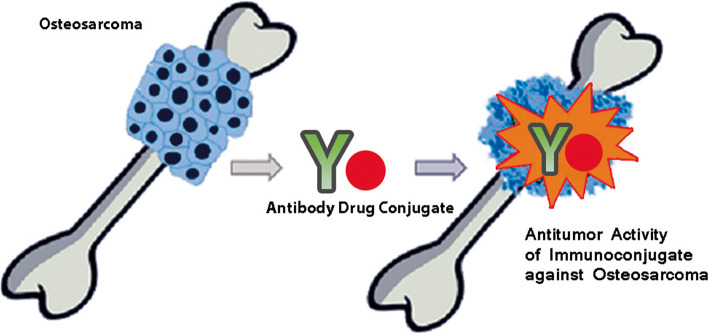
OS targeting by antibody-drug conjugates wherein the cytotoxic agents and other therapeutics are linked to antibody designed against specific cell target (OS) [72]

In immunoconjugates, the monoclonal antibodies (mAb) are conjugated to a drug or to the surface of drug-loaded nanocarriers to target them at OS (Fig. 3) [72]. The fully human antibodies are developed to minimize the immunogenicity. The immunoconjugates are prepared for pharmacological agents, toxins, and radionuclides [73–75]. The antibodies are conjugated with therapeutic moiety by a chemical linker which may be breakable or non-breakable [76]. The cytotoxic efficacy, pharmacokinetic performance, and toxicity of conjugated drugs depend mainly on the nature of the linker; therefore, the selection of a suitable linker is imperative in designing immunoconjugates [77]. Recently, it is reported that pharmacological agents that block tubulin polymerization are suitable candidates for the preparation of immunoconjugates. Of the toxins, bacterial toxins such as Pseudomonas exotoxin A and diphtheria toxin are mainly used to make immunoconjugates. The chief limitation of using plant and bacterial origin toxins is that repeated administration results in the blocking of production of anti-toxin antibodies. The radioactive isotopes of iodine and yttrium belong to the radionuclides class are appropriate for immunoconjugation [78]. Michael et al. developed glembatumumab vedotin immunoconjugate (GVIC) for targeting osteosarcoma. The GVIC showed remarkable cytotoxicity against osteosarcoma cells [79]. In another study, Anderson and the group have developed hemotoxin (pokeweed antiviral protein) TP-3 (IgG2b mAb) (HTP) immunoconjugate for osteosarcoma treatment. HTP demonstrated significant cytotoxic activity against OHS osteosarcoma [80].

The cell surface receptor-activated leukocyte adhesion molecule (ALCAM, CD166), a transmembrane glycoprotein from the immunoglobulin family, can be used to target OS. Recently, Federman and group have designed anti-CD166 mAb conjugated to doxorubicin-loaded liposomal nanoparticles against OS. These antibody-targeted liposomes demonstrated incredible cytotoxicity against OS cells than non-targeted liposomes [81]. However, the concept of immunoconjugation has faced a few challenges such as low therapeutic index and cross-reactivity with normal tissues. Besides, the pharmacokinetic characteristics of the therapeutic material can be changed following conjugation resulting in toxicity towards various organs [65].

#### Bisphosphonates (BP)-drug conjugates

Bisphosphonates (BP) are similar to endogenous pyrophosphate possessing a greater affinity for hydroxyapatite (HA). BP is commonly used in the treatment of bone diseases such as osteoporosis, Paget’s disease, benign, and malignant bone diseases [82–85]. The oxygen atom in pyrophosphate (P-O-P) when replaced with carbon atom (P-C-P) results in improved stability against chemical and enzymatic degradation [83, 84]. The high affinity of BP towards HA helps to block osteoclast recruitment and adhesion to the bone matrix decreases osteoclast half-life and inhibits its activity directly [85]. In radiotherapy, BP is now used as a targeting agent. The bisphosphonate radio-labeled-drug conjugates are employed as particular markers in imaging bone pathologies [86–88].

The BP-drug conjugate is found to be a potent and promising approach in drug delivery due to noteworthy characteristics of BP including its high affinity towards HA and potency in osteoporosis treatment. The different therapeutics such as small molecule drugs, macromolecules, and imaging agents can be conjugated with BP for bone targeting [89–94]. The important benefits offered by BP-drug conjugates are improved bone targeting thereby reduced systemic toxicities, and increased circulation half-life. Katrin and the group have fabricated a bisphosphonate prodrug of doxorubicin for targeting bone metastasis. The prodrug displayed a fast release of DOX and adequate stability over several hours in human plasma [95]. Similarly, Rudnick-Glick and co-workers have developed doxorubicin-conjugated BP NPs (DCBNPs) for targeting primary and metastatic bone tumors. The DCBNPs have demonstrated improved efficacy and targeting in both Soas-2 human osteosarcoma xenograft mouse model and orthotopic bone metastases mCherry-labeled 4T1 breast cancer mouse model [67].

Despite the various advantages, the several limitations associated with the use of BP. The one of the key limitations is that they target the bone very efficiently than OS specifically. Besides, the prolonged residence of BP in the bone tissue may inhibit osteoclasts and bone homeostasis resulting in osteonecrosis of the jaw, nephrotoxicity, hypocalcemia, and ocular dysfunction. Furthermore, BP has low oral bioavailability (< 1%) causing irritation of the gastrointestinal (GI) tract [71].

#### HPMA-drug conjugates

HPMA [N-(2-hydroxypropyl)-methacrylamide] is a hydrophilic, non-toxic, and non-immunogenic copolymer used for bone targeting in osteoporosis [96]. The in vivo clearance of this copolymer occurs primarily through the renal glomerular filtration because of its water solubility [97]. HPMA copolymers can be used as carriers for conjugating drug molecules. These conjugations offer various advantages including improved water solubility of hydrophobic drugs; protection of the drug from premature metabolism before its distribution to the tissue targets, enhanced circulation half-life, and bioavailability. However, the vital challenge associated with this approach is the low therapeutic carrying capacity [97].

The HPMA-drug conjugate (prodrug) can efficiently target solid tumors (OS) by a passive targeting mechanism [98]. Besides, multiple therapeutics and targeting moieties can be introduced using the HPMA copolymer. The absorption of HPMA copolymer conjugates generally occurred through the endocytosis mechanism [99]. The surface decoration of HPMA-drug conjugates with specific targeting ligand can help to improve the organ specificity and the rate of their cellular uptake [100, 101]. The better efficacy and safety profile, and well-established conjugation chemistry has shown the promising potential of these conjugates in drug delivery to the skeleton. Ehud et al. have synthesized aminobisphosphonate alendronate (ABA) and TNP-470 (a potent anti-angiogenic agent) conjugate with HPMA copolymer through a glycine-proline-norleucine linker. This link is cleaved by a cysteine protease overexpressed (cathepsin K) at resorption sites in bone tissues. In their study, both passive and active targeting is achieved. The in vitro cytotoxicity study using Saos-2 and MG-63-Ras human osteosarcoma cells has shown significant cell growth inhibition in presence of the conjugate [98].

#### Tetracycline-drug conjugates

The tetracycline (TC), which is an antibiotic having high affinity and binds strongly to bone [67], can bind to the bone apatite through the formation of chelates with calcium [102]. Besides, it causes inhibition of bone resorption as well as collagenases. Moreover, it decreases the production of acid and the secretion of lysosomal cysteine proteinases. Another important characteristic of TC is the enhancement of active osteoblasts by enhancing the expression of procollagen mRNA [103, 104]. It can be conjugated with different therapeutics for the treatment of a variety of skeletal diseases due to their osteotropicity. Wang and the group have observed improved bone targeting of drug delivered using PLGA-NPs wherein the TC is covalently bonded to PLGA. This improved bone targeting is due to the interaction between TC with HA [105]. Despite the above major benefits, the main challenges associated with the use of TC for conjugation with the drug is its complicated chemical structure and its poor stability during chemical modification.

### Advanced approaches

#### Gene therapy

Based on the pathogenesis and genetic abnormities of OS, there is a need to explore new potential molecular targets. Gene delivery using viral and non-viral vectors have been developed where gene (DNA) is delivered into human cells. The gene delivery using viral vectors involves the use of a genetically modified virus. The different viral vectors used for gene delivery in OS include adenoviruses, adeno-associated viruses, herpes simplex viruses, lentiviruses, and retroviruses. Ganjavi and co-workers have studied the effect of adenovirus-mediated p53 gene (Ad-wtp53 and Ad-mutp53) transfer in different OS cell lines including Saos-2, HOS, KHOS/NP, and MNNG. The MTT assay demonstrated a dose-dependent decrease in cell viability with Ad-wtp53 after 72 h post-treatment [106].

DNA transfer by non-viral vectors can use either liposome (phospholipid-DNA complex, lipoplex) or DNA-polymer complex (Polyplex). In the case of liposomal gene delivery, the surface charge, size, and morphology can be engineered to achieve the desired performance [107]. Among the different nanocarriers, currently, micelleplex has gained the attention of the researchers due to its various noteworthy features like simple preparation methods, more stability, and minimum toxicity [108]. Mariana et al. have fabricated micelleplexes loaded with miR-145 for osteosarcoma treatment. The treatment with micelleplex has resulted in significantly increased cell death [109]. The main benefit of the use of non-viral vectors is that they are safe to administer. However, very little transfection efficiency and rapid inactivation in contact with serum are the chief limitations. Besides, poor pharmacokinetics, cellular toxicity, and drug resistance are the chief challenges in the treatment of OS using gene therapy [110].

#### Gene-directed enzyme prodrug therapy (GDEPT)

It is also recognized as suicide gene therapy or molecular chemotherapy. It is based on the capacity of the gene product of a transduced cell to convert a non-toxic prodrug into a toxic compound. The chief benefit of this strategy is that not every single tumor cell needs to express this product to eradicate a tumor population, due to the so-called bystander effect. Various prodrug-converting enzymes such as herpes simplex thymidine kinase, bacterial cytosine deaminase, carboxylesterase-2 have been studied for OS [111]. Dinja et al. have developed gene-directed enzyme prodrug therapy using carboxylesterase-2 of the human liver and Camptothecin-11 (cytotoxic agent). The in vitro study exhibited increased sensitivity of Camptothecin-11 (up to 70 fold) against primary OS cultures obtained from high-grade OS suffering patients after its transduction with adenoviral vector (Ad-sCE2) [112]. The different challenges that have limited the clinical applications of GDEPT include natural metabolism of the prodrugs by the liver enzymes causing serious toxicity to various organs (neurotoxicity, nephrotoxicity, cardiotoxicity, etc.) [13].

#### T cell therapy

It is one of the potentials and alternative approaches used to treat the OS when conventional and other therapies are failed. Besides, it is useful to minimize the difficulties in the treatment. In this strategy, the conventional antigen-presenting cells are used to produce tumor-specific T cells ex vivo. However, the main drawback of this strategy is more time requirement, much less frequency for tumor-specific T cells, and sensitivity to the immunosuppressive tumor microenvironment. Therefore, antigen (tumor-associated antigens, TAA)-specific genetically modified T cells could be a promising approach to overcome the above problems [113]. The various TAA associated with OS include human epidermal growth factor receptor 2 (HEGR2) [114, 115], interleukin 11 receptor alpha (IL11Rα) [116], melanoma-associated antigen (MAG) and g melanoma antigen (GAGE) family members [117], GD2 (a disialoganglioside; not a protein tumor-associated antigen) [118], New York esophageal squamous cell carcinoma 1 (NY-ESO-1) [117], clusterin-associated protein 1 (CLUAP1) [119], papillomavirus binding factor [120], fibroblast activation protein (FAP) [121], and tumor endothelial marker 1 (TEM1) [122], and B7-H3 [123]. Wang and co-workers have treated the OS using anti-CD166/4-1BB chimeric antigen receptor T cell therapy. In vitro studies have shown the killing of OS cells using CD166.BBζ CAR-T cells. Moreover, CD166.BBζ CAR-T cells demonstrated the regression of tumors with no noticeable toxicity upon intravenous injection into mice. However, the significant challenges associated with T cell therapy are limited T cell expansion and lack of tackling the inhibitory tumor micro-environment [124].

## Conclusions

The OS treatment is challenging because of unknown etiology, lack of genetic stability, greater histological heterogeneity, and lack specific biomarkers [118]. Moreover, diverse barriers (tumor microenvironment, tumor vasculature, stromal cells) are also limiting drug delivery applications in OS.

Despite the extensive advancement in drug delivery to OS, there is a prerequisite to ascertain the in vitro and in vivo performance of developed novel drug delivery systems using a variety of animal models. Animal models are of vital importance for the proper understanding of the genetic basis of OS. Besides, they play a crucial role in advancing preclinical studies intended for the rational expansion of novel therapeutic approaches and their validation of preceding clinical trials. Accurate recapitulation of the natural course of the disease is one of the prerequisites of any animal model for human disease. In the case of OS, the etiology and pathogenesis are not completely known; therefore, the endowment and induction of representative experimental models are challenging and incomplete [125].

The nanoformulations and other advanced approaches developed against OS were mostly evaluated using in vitro tissue cultures. One of the key limitations of the transformed OS cell line is a lack of correct representation of the human condition. Besides, the OS cell lines have displayed distorted gene expression during the in vitro culture studies. The other limitations of OS tissue culture include they are expensive and required a long time for tumor initiation, tumor progression, and treatment response. Moreover, some OS cell cultures could not fulfill the need for an in vivo metastatic model [126–128].

The long-term biosafety evaluation of several NCs and other advanced approaches using more relevant animal models is of critical importance, and challenging [129]. The other critical evaluations of nanomaterials, targeting materials, and other advanced approaches developed against OS are not well-developed for use in OS patients. Moreover, the approaches developed are still at the cellular and animal experimental phase and may take more time for the clinical application.

A huge research development has been done in bone-targeted drug delivery including active targeting approaches having a high affinity for HA present on the bone surface. Further, gene therapy alone or in combination with chemotherapy, radiation therapy, and conventional surgery could be a future potential approach for the treatment of OS. In addition, the genetic modification of T cells and combining it with other therapies such as chemotherapy, and radiotherapy could be another promising and potential approach for the effective treatment of OS. More importantly, combining the nanoparticulate approach for gene and T cell delivery alone or in combination with other approaches could further improve the therapeutic outcomes in OS treatment.

## Data Availability

All data and material are available upon request.

## Abbreviations

ANPs: Aragonite nanoparticles
ALD: Alendronate
ATRA: All-trans retinoic acid
BP: Bisphosphonates
CUR: Curcumin
CeO2NPs: Cerium dioxide microparticles
DTX: Docetaxel
DOX: Doxorubicin
EPR: Enhanced permeability and retention
HA: Hydroxyapatite
HEGR2: Human epidermal growth factor receptor 2
HPMA: N-(2-hydroxypropyl)-methacrylamide
HRML: Hyaluronidase-responsive multi-layer liposome
ION: Iron oxide nanocage
MAG: Melanoma-associated antigen
mAb: Monoclonal antibodies
MDR: Multi-drug resistance
MTX: Methotrexate
NA: Nucleic acid
NCs: Nanocarriers
NPs: Nanoparticles
NL: Nanoliposomes
OS: Osteosarcoma
PMs: Polymeric micelle
PDPN: Peptide-decorated polypeptide nanogel
PEG: Polyethylene glycol
P-O-P: Pyrophosphate
RES: Reticulo-endothelial system
TC: Tetracycline
SAL: Salinomycin
TAA: Tumor-associated antigens
